# Genome-Wide Identification of Differentially Expressed Genes Associated with the High Yielding of Oleoresin in Secondary Xylem of Masson Pine (*Pinus massoniana* Lamb) by Transcriptomic Analysis

**DOI:** 10.1371/journal.pone.0132624

**Published:** 2015-07-13

**Authors:** Qinghua Liu, Zhichun Zhou, Yongcheng Wei, Danyu Shen, Zhongping Feng, Shanping Hong

**Affiliations:** 1 Research Institute of Subtropical Forestry, Chinese Academy of Forestry, Fuyang, Zhejiang, People’s Republic of China; 2 Zhejiang Provincial Key Laboratory of Tree Breeding, Fuyang, Zhejiang, People’s Republic of China; 3 Laoshan Forest Farm of Chunan County, Chunan, Zhejiang, People’s Republic of China; University of Copenhagen, DENMARK

## Abstract

Masson pine is an important timber and resource for oleoresin in South China. Increasing yield of oleoresin in stems can raise economic benefits and enhance the resistance to bark beetles. However, the genetic mechanisms for regulating the yield of oleoresin were still unknown. Here, high-throughput sequencing technology was used to investigate the transcriptome and compare the gene expression profiles of high and low oleoresin-yielding genotypes. A total of 40,690,540 reads were obtained and assembled into 137,499 transcripts from the secondary xylem tissues. We identified 84,842 candidate unigenes based on sequence annotation using various databases and 96 unigenes were candidates for terpenoid backbone biosynthesis in pine. By comparing the expression profiles of high and low oleoresin-yielding genotypes, 649 differentially expressed genes (DEGs) were identified. GO enrichment analysis of DEGs revealed that multiple pathways were related to high yield of oleoresin. Nine candidate genes were validated by QPCR analysis. Among them, the candidate genes encoding geranylgeranyl diphosphate synthase (GGPS) and (-)-alpha/beta-pinene synthase were up-regulated in the high oleoresin-yielding genotype, while tricyclene synthase revealed lower expression level, which was in good agreement with the GC/MS result. In addition, DEG encoding ABC transporters, pathogenesis-related proteins (PR5 and PR9), phosphomethylpyrimidine synthase, non-specific lipid-transfer protein-like protein and ethylene responsive transcription factors (ERFs) were also confirmed to be critical for the biosynthesis of oleoresin. The next-generation sequencing strategy used in this study has proven to be a powerful means for analyzing transcriptome variation related to the yield of oleoresin in masson pine. The candidate genes encoding GGPS, (-)-alpha/beta-pinene, tricyclene synthase, ABC transporters, non-specific lipid-transfer protein-like protein, phosphomethylpyrimidine synthase, ERFs and pathogen responses may play important roles in regulating the yield of oleoresin. These DEGs are worthy of special attention in future studies.

## Introduction

Oleoresin, as a mixture of many terpenoids including turpentine (monoterpenes and sesquiterpenes) and rosin (diterpenes), is stored in resin ducts or blisters in stems, roots, needles and cones of conifer trees [1]. Terpenoids as an important natural source for industrial chemicals were used widely in solvents, cleaning agents for varnishes and paints, flavors and fragrances, synthetic rubber, disinfectants, coatings, printing ink resins, and waterproofing materials [2–5]. Terpenoids also can be exploited as advanced liquid biofuels. Biofuels derived from terpenoids have similar properties as diesel and gasoline, and the density and hygroscopicity are amenable to mixing with fossil fuels [6]. In addition, terpenoids also play an important role in defense system of conifer trees. The resin ducts or blisters are severed and oleoresin is released after suffering abiotic or biotic stimuli such as mechanical wounding, insect attack and pathogen invasion. Emitted monoterpenes and sesquiterpenes are toxic volatiles that can directly affect herbivores, while diterpene resin acids provide the protection through forming physical barriers at the point of wound [7–8]. Other studies have found that the trees releasing a large amount of oleoresin upon wounding exhibit the strongest resistance to beetle attack in southern pines [9].

In addition to constitutive oleoresin, new oleoresin can be synthesized and traumatic resin ducts (TRD) can be formed after stem suffering abiotic or biotic stimuli in conifers [10–11]. In conifers, the yield of oleoresin is a quantitative trait under moderate to strong genetic control [12–14]. Important genetic gains can be obtained through the selection of high resin yielders [15]. Based on the traditional breeding, many high oleoresin-yielding families, clones and individuals have been selected [16–18]. Previous studies demonstrate that the yield of oleoresin is strongly correlated with tree diameter, percentage of live crown, radial resin canal number and resin canal volume [15,19]. The relationship between the yield of oleoresin and the concentration of each terpenoid is also investigated and the results show that some terpenoids can be used as the diagnostic marker for high oleoresin-yielding trees [20]. However, molecular studies on genes related to the yield of oleoresin in conifers are still rare. Exploration of the genes for regulating the yield of oleoresin can reduce the breeding cycle and rapidly increase the production of oleoresin in stems of conifers by genetic engineering.

As its well known, the biosynthesis of oleoresin is usually completed through two separate pathways of methyl-erythritol 4-phosphate (MEP) and mevalonate (MVA) pathways in conifers. Monoterpenes and diterpenes are biosynthesized by MEP pathway [21], while the biosynthesis of sesquiterpenes is completed by MVA pathway [22]. During the biosynthesis of oleoresin terpenoids, the enzymes for biosynthesis include 1-deoxyxylulose 5-phosphate reductoisomerase (DXR), prenyltransferases, 1-deoxyxylulose 5-phosphate synthase (DXS), hydroxymethylbutenyl 4-phosphate reductase (HDR), terpenoid synthase (TPS) and cytochrome P450-dependent monooxygenase. In recent decades, many studies on terpenes have focused on the potential role in the resistance against abiotic or biotic stimuli. TPS enzymes as the key enzymes for generating diverse terpenoids have been explored widely. Many TPS genes associated with the resistance have been identified in several coniferous species including loblolly pine (*P*. *taeda*), grand fir (*Abiesgrandis*), and spruce species [23–25]. The genes encoding TPS of genus *Pinus* belong to TPS*d* gene families [1].

Masson pine (*P*. *massoniana*) is an important timber and resource for oleoresin in South China. Approximately 90% of rosin comes from masson pine. Oleoresin is annually tapped using the bark streak method of wounding, and collected from living pine trees after the diameter at breast height (DBH) reaching a certain size class in China. The State Forestry Administration of China stipulates that pine trees are not used for the tapping of oleoresin until the DBH exceeds 20 cm. The yield of oleoresin varies greatly within masson pine [15]. Many superior provenances, families, and clones with high yield of oleoresin have been selected in the progress of genetic improvement [16–18]. However, the genetic mechanisms for regulating the yield of oleoresin are unknown.

The next-generation sequencing has greatly contributed to biological studies in non-model systems. The *de novo* transcriptomic analysis has been used to explore the differential gene expression profile among tissues subjected to different treatments. In the present study, the aim is to explore the transcriptome of secondary xylem in masson pine and identify the genes with differential expression between the high and low oleoresin-yielding trees based on high-throughput sequencing technology. These results will be valuable for genomic and genetic studies on high oleoresin-yielding masson pine in the future.

## Results

### Compositional characterization of oleoresin compounds in masson pine

In order to reveal the compositional properties of oleoresin, we performed gas chromatography-mass spectrometry (GC/MS) analysis for high and low oleoresin-yielding masson pines. Totally 26 oleoresin terpenoids with matching degree more than 90% to NIST and the content higher than 0.01% were identified. As shown in Table 1 and S1 Fig, 26 oleoresin terpenoids were characterized by qualitative and quantitative analysis, including 7 monoterpenes, 11 sesquiterpenes and 8 diterpenes. For both high and low oleoresin-yielding genotypes, the most abundant terpenoid is alpha-pinene, accounting for 85% of all monoterpenes, which is followed by beta-pinene and limonene. Among sesquiterpenes, the most abundant compounds are longifolene and caryophyllene, and the sum of them reaches up to 82% of sesquiterpenes. Among dierpenes, palustric acid reveals the highest level, which is followed by neoabietic acid and abietic acid.

**Table 1:** The average content and SD of oleoresin compounds and significant difference between 35 high and 35 low oleoresin-yielding masson pines using non-parametric Mann-Whitney test.

		High oleoresin-yielding trees		Low oleoresin-yielding trees		
Compounds	RI	Mean (mg/g)	SD	Mean (mg/g)	SD	Sig.
Monoterpenes		170.53	18.97	173.79	18.96	0.5076
Tricyclene	920	0.27	0.08	0.34	0.15	0.0270*
alpha-Pinene	932	146.19	16.49	149.71	16.43	0.4100
Camphene	944	4.16	0.71	3.69	0.64	0.0091**
beta-Pinene	973	9.75	3.05	10.95	4.15	0.2075
beta-Myrcene	991	3.12	0.6	2.75	0.55	0.0146*
Limonene	1025	5.51	1.07	4.74	1.44	0.0219*
Borneol	1153	0.98	0.4	1.03	0.57	0.7040
Sesquiterpenes		130.48	31.31	106.76	36.68	0.0092**
Longipinene	1344	5.85	2.56	4.32	1.98	0.0122*
Longicyclene	1363	5.31	2.14	4.97	2.55	0.5790
Ylangene	1368	0.79	0.32	0.66	0.31	0.1175
Copaene	1372	0.14	0.08	0.14	0.11	0.8415
Sativene	1383	2.8	1.14	2.58	1.33	0.4925
Longifolene	1397	88.38	23.02	66.47	26.37	0.0011**
Caryophyllene	1413	19.79	6.87	21.57	8.73	0.3826
Humulene	1448	3.4	1.24	3.62	1.71	0.5681
b-Farnesene	1458	1.66	1.1	1.38	1.16	0.3394
Germacrene D	1476	2.01	2.95	0.79	0.99	0.0951
δ-Cadinene	1521	0.35	0.25	0.27	0.19	0.1878
Dierpenes		523.85	24.66	540.84	25.65	0.0114*
Pimaric acid	2219	49.5	12.06	53.73	7.61	0.1095
Sandaracopimaric acid	2227	11.15	1.43	11.35	1.54	0.6039
Isopimaric acid	2248	21.93	10.86	15.91	12.2	0.0481*
Palustric acid	2256	268.59	31.12	268.15	27.96	0.9541
Dehydroabietic acid	2273	35.58	12.97	38.61	13.53	0.3803
8,12-Abietadienoic acid		1.89	1.1	1.66	1.05	0.4228
Abietic acid	2293	57.66	10.47	69.31	13.66	0.0005**
Neoabietic acid	2341	77.55	9.15	82.11	11.23	0.0899

* and ** show the significant difference at p value less than 0.05 and 0.01, respectively.

Using the non-parametric Mann-Whitney test, the contents of 8 oleoresin terpenoids including tricyclene, camphene, limonene, beta-myrcene, longipinene, longifolene, isopimaric acid and abietic acid, revealed significantly difference between high and low oleoresin-yielding genotypes. For camphene, beta-myrcene, limonene, longipinene, longifolene and isopimaric acid, the contents of these compounds were higher in high oleoresin-yielding trees, while the contents of tricyclene and abietic acid were higher in low oleoresin-yielding trees. Overall, the high oleoresin-yielding trees had a higher content of sesquiterpene and lower content of diterpene when compared with the low oleoresin-yielding trees.

### RNA-Seq and *de novo* transcriptome assembly

In order to obtain information in xylem tissues of masson pine at major oleoresin-secreting stages, total RNA was isolated from twenty high and low oleoresin-yielding clones for RNA-Seq performed on the Illumina Hiseq2000 platform. After quality assessment and data screening, we obtained 40,690,540 high quality reads (8.2 gigabase pairs) with 84.80% (Q≥30 bases). Then these reads were assembled into 3,641,300 contigs using SOAPdenovo (S1 Table). In the following, using Trinity assembly program, short-read sequences were assembled into 137,499 transcripts with a mean length of 904 bp. Finally, 84,842 unigenes were obtained with a mean length of 690 bp. Among these unigenes, 15,713 unigenes (18.52%) were larger than 1 kb. The results suggested that the high quality and throughput sequences were prepared for the further analysis. The length distributions of transcripts and unigenes (S1 Table) have the similar tendency with N50 values of 1,671 bp and 1,291 bp, respectively.

### Functional annotation

Functional annotation of retrieved unigenes was carried out by aligning sequence similarity with several public databases with a threshold of E-value less than 10^-5^, and the annotation results are listed in S2 Table. A total of 35,353 (41.7%) unigenes were annotated in the public databases (S3 Table). Based on the annotation using the NCBI non-redundant protein (Nr) database, 27,147 unigenes were distributed into one or more GO terms, with 28.36% for cellular components, 22.80% for molecular functions and 48.84% for biological processes (S2 Fig). In addition, 9,990 of 84,842 sequences could be assigned to Cluster of Orthologous Groups of proteins (COG) database for functional prediction and classification (S3 Fig).

### Analysis of differentially expressed genes (DEGs)

To screen the DEGs related to yield of oleoresin in masson pine, 10 high and 10 low oleoresin-yielding genotypes were used for the establishment of six sequencing libraries. The six libraries generated 9.61–12.40 million high-quality reads. Subsequently, the sequences of these reads were mapped to the transcriptome database described above. More than 80% of these reads matched the sequences in the transcriptome, and 92% of these reads could be identified unequivocally by the unique mapping (S4 Table). The reads were mapped to unigenes and quantified to obtain the abundance of gene expression by Reads per Kilobase per Million mapped Reads (RPKM). In order to explore molecular difference in expression levels between high and low oleoresin-yielding masson pines, the significant DEGs were compared by applying cutoff of p-value less than 0.05 (FDR BH corrected). A total of 649 DEGs were identified (S5 Table), and 343 unigenes were significantly up-regulated and 306 unigenes were significantly down-regulated. These results suggested that the high yield of oleoresin might be due to the different expression levels of these DEGs.

### Multiple pathways are involved in the high yielding of oleoresin identified by GO enrichment analysis of DEGs

**Fig 1:**
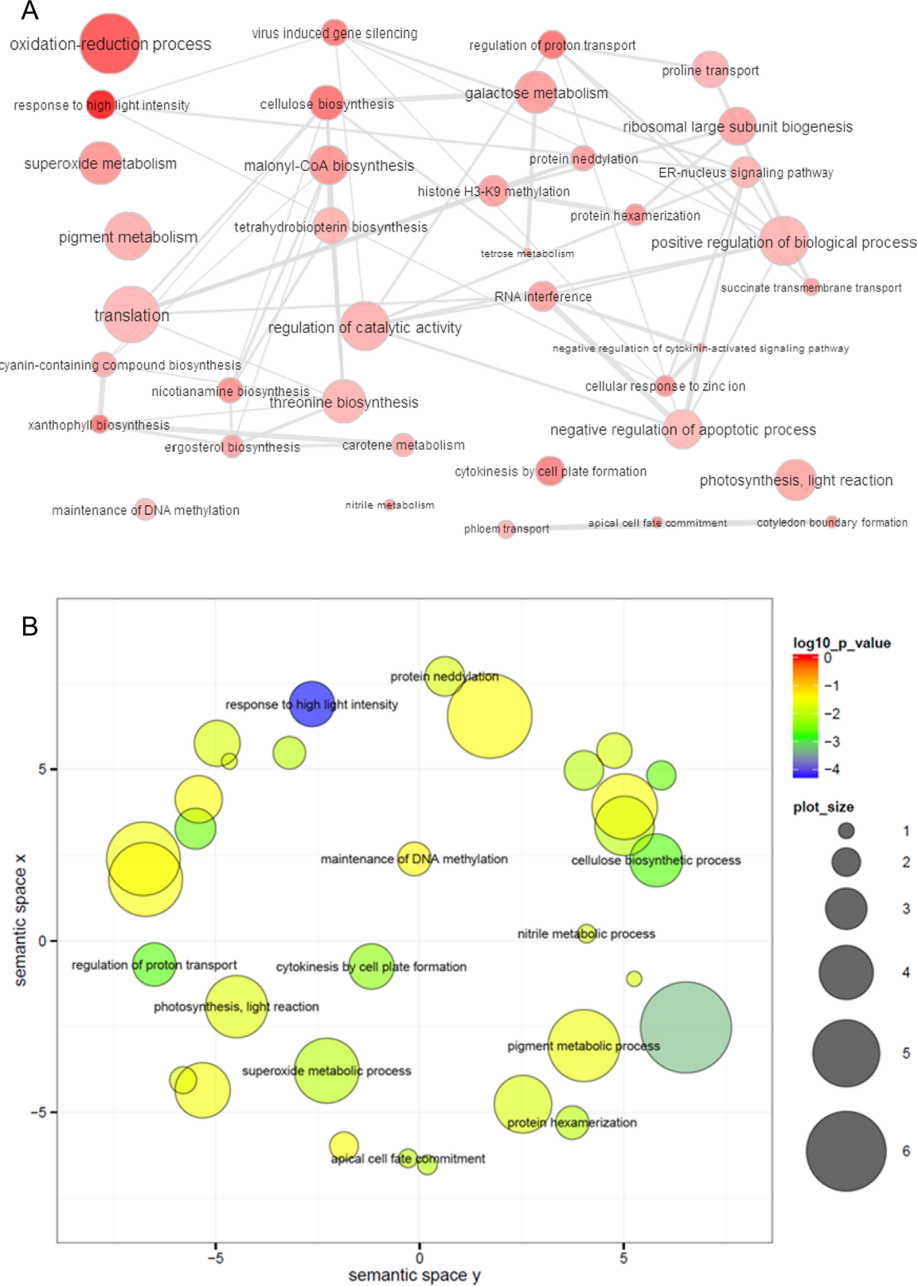
GO enrichment analysis of DEGs between high and low oleoresin-yielding masson pines using REVIGO. **(A)** The interactive graph of the enriched GO terms for DEGs. Bubble color indicates the p-value; bubble size indicates the frequency of the GO term. Highly similar GO terms are linked by edges in the graph, where the line width indicates the degree of similarity. (**B**) The scatter plot showing the significance of the GO term for DEGs (terms remaining after reducing redundancy) in a two-dimensional space derived by applying multi-dimensional scaling to a matrix of GO term semantic similarities. Bubble color indicates the p-value for the false discovery rates. The circle size represents the frequency of the GO term (more general terms are represented by larger size bubbles).

To achieve a functional annotation of the genes related to the yield of oleoresin, we performed GO enrichment analysis of 649 DEGs. In total, we uncovered 109 GO terms that were enriched significantly (*p* < 0.05, FDR corrected) (S6 Table). In the biological process, the enriched GO terms for DEGs were “oxidation-reduction process”, “regulation of cellular biosynthetic process”, “cellular protein metabolic process”, “regulation of nitrogen compound metabolic process” and “translation” (Fig 1A, S6 Table). However, the most significant GO terms between high and low oleoresin-yielding trees were “response to high light intensity”, “oxidation-reduction process” and “response to far red light”. The enriched GO terms were also visualized by ReviGO [26] (Fig 1B), and pathways such as light response, photosynthesis, and pigment metabolism (Fig 1B), indicating that multiple biological pathways might be involved in regulating the yield of oleoresin.

### Validation of candidate gene expression by qRT-PCR

**Fig 2:**
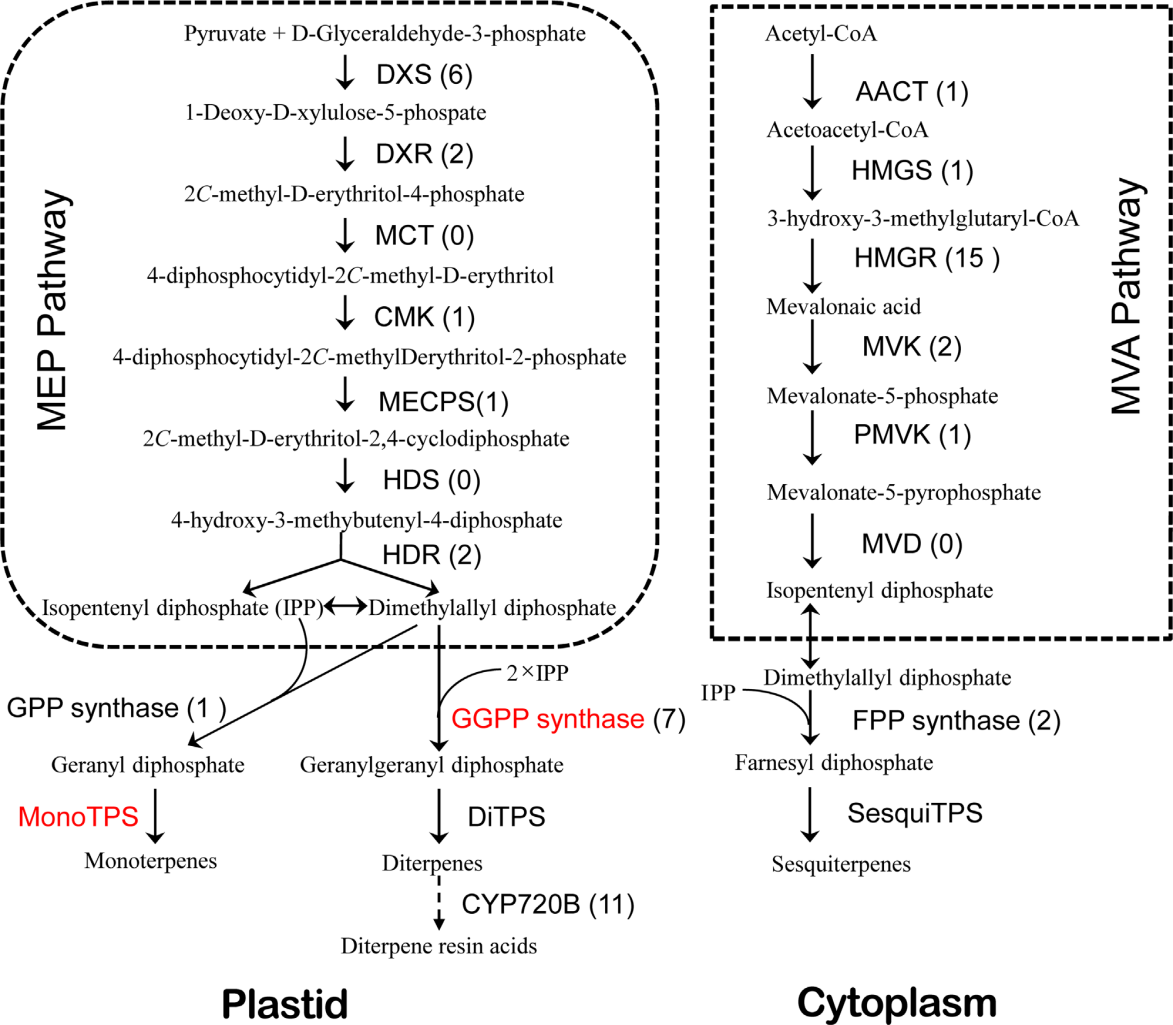
Biosynthetic pathway of terpenoids in *P*. *massoniana* (adapted from Zulak et al [22]). The number of unigenes homologous to gene families encoding these enzymes was provided in parentheses. The enzymes highlighted with red fonts showed that DEGs were detected in gene families encoding these enzymes. DXS: 1-deoxy-dxylulose-5-phosphate synthase; DXR: 1-deoxy-D-xylulose-5-phosphate reductoisomerase; MCT: 4-diphosphocytidyl-2Cmethyl-D-erythritol synthase; CMK: 4-diphosphocytidyl-2C–methyl-D-erythritol kinase; MECPS: 2C-methyl-D-erythritol 4-phosphate cytidylyltransferase; HDS: 1-hydroxy-2-methyl-2-(E)-butenyl-4-diphosphate synthase; HDR: 1-hydroxy-2-methyl-2-(E)-butenyl-4-diphosphate reductase; AACT: acetoacetyl-CoA thiolase; HMGS: 3-hydroxy-3-methylglutaryl-CoA synthase; HMGR: 3-hydroxy-3-methylglutaryl-CoA reductase; MVK: mevalonate kinase; PMVK: phosphomevalonate kinase; MVD: mevalonate diphosphate decarboxylase; IPPI: isopentenyl-diphosphate isomerase; FPPS: farnesyl diphosphate synthase; GPPS: geranyl diphosphate synthase; SesquiTPS: Sesquiterpene synthase; MonoTPS: Monoterpene synthase; DiTPS: Diterpene synthase; CYP720B: Abietadienol/abietadienal oxidase PtAO.

In MEP and MVA pathways, 96 unigenes were associated with the biosynthesis of terpenoid backbone according to transcriptome database. Except 4-diphosphocytidyl-2Cmethyl-D-erythritol synthase (MCT), mevalonate diphosphate decarboxylase (MVD) and 1-hydroxy-2-methyl-2-(E)-butenyl-4-diphosphate synthase (HDS), those enzymes participating the biosynthesis of terpenoid backbone in MVA and MEP pathways had one or more corresponding unigenes (Fig 2). Among them, 43 unigenes annotated as TPS by Nr or Swissprot database were obtained, including 14 unigenes for monoTPS, 19 unigenes for sesquiTPS and 10 unigenes for diTPS.

**Fig 3:**
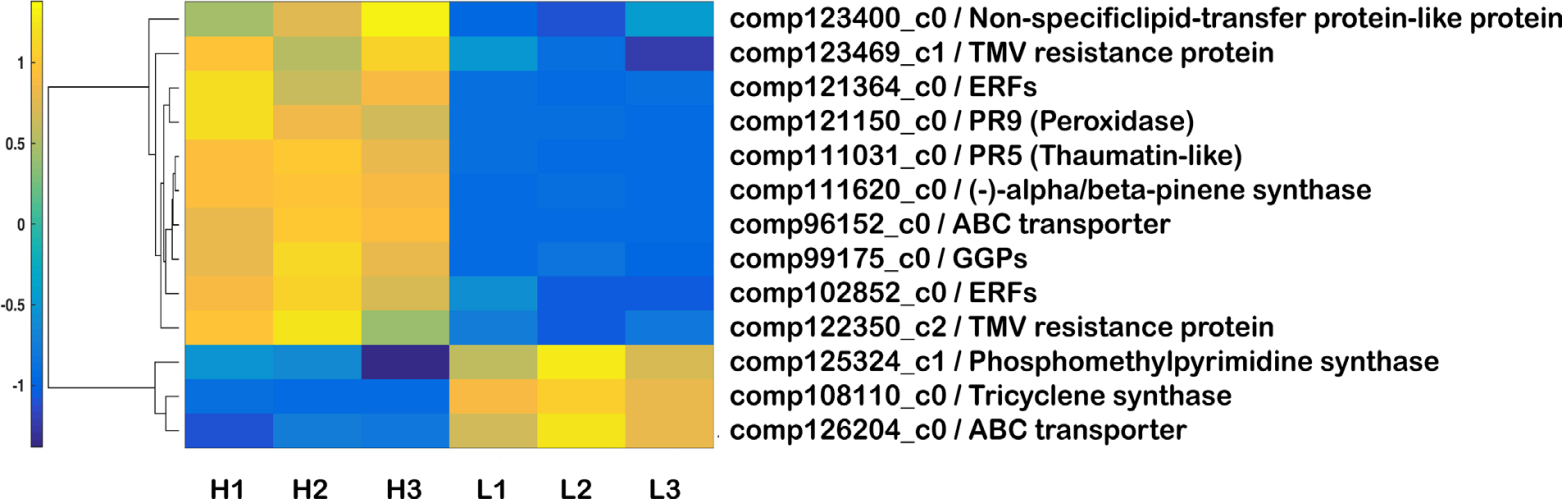
Clustering and heatmap of key DEG related to the yield of oleoresin based on their expression levels. Three biological replicates were used for high-yielding genotype (H1, H2, H3) and low-yielding genotype (L1, L2, L3). Colored bars on the left of the heatmap mark the major distinct branches in the clustering tree grouping genes with similar expression pattern.

The biosynthesis pathway of terpenoid backbone played central roles in the biosynthesis of oleoresin. Hierarchical clustering of these candidate genes was shown in Fig 3. In high oleoresin-yielding trees, the expression of GGPS and (-)-alpha/beta-pinene synthase was approximately 8 and 14 times higher than that in low oleoresin-yielding trees, respectively. However, the expression of tricyclene synthase was approximately 87 times higher than that in high oleoresin-yielding trees. In addition, many DEGs coding phosphomethylpyrimidine synthase, non-specific lipid-transfer protein-like protein, ethylene responsive transcription factors (ERFs), ATP binding cassette (ABC) transporter, pathogenesis-related proteins (PR5 and PR9) and TMV resistance protein had obvious different expressions between high and low oleoresin-yielding trees. Among these DEGs, two candidate genes of ERFs, two candidate genes of TMV resistance protein, two candidate genes of PRs, one candidate gene of ABC transporter and one candidate gene of non-specific lipid-transfer protein-like protein were up-regulated in high oleoresin-yielding trees (S7 Table). One candidate gene of phosphomethylpyrimidine synthase and one candidate gene of ABC transporter were down-regulated in high oleoresin-yielding trees (S7 Table).

**Fig 4:**
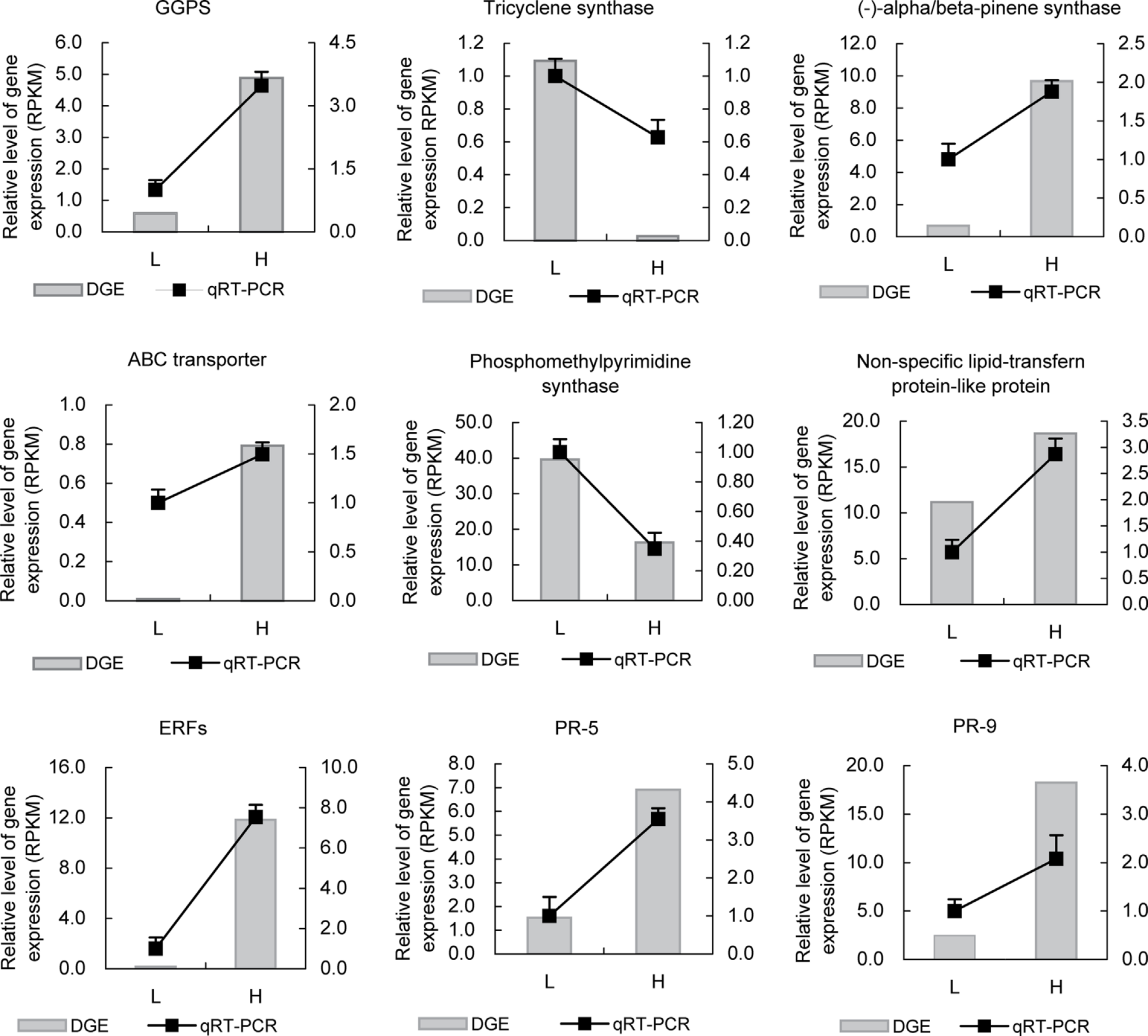
Quantitative RT-PCR validation of tag-mapped candidate genes associated with the yield of oleoresin. Relative expression levels of qRT-PCR calculated using Elongation factor 1-alpha as the internal control were shown in the right y-axis. Relative expression levels of DGE were shown in the left y-axis.

To validate the reliability of the results from DEGs, we selected 9 representative candidate genes for qRT-PCR analysis (Fig 4). Among 9 candidate genes, three candidate genes involve in the biosynthesis of terpeniods including GGPS (comp99175_c0), (-)-alpha/beta-pinene (comp111620_c0) and tricyclene synthase (comp108110_c0), and two candidate genes of PR5 (comp111031_c0) and PR9 (comp121150_c0) involve in defense response. The remaining four candidate genes encoded ABC transporter (comp96152_c0), phosphomethylpyrimidine synthase (comp125324_c1), non-specific lipid-transfer protein-like protein (comp123400_c0) and ERFs (comp121364_c0). Overall, the results of qRT-PCR analysis were consistent with DEG analysis, suggesting that these candidate genes might be the important candidates for controlling the yield of oleoresin (Fig 4).

## Discussion

It is known that the yield of oleoresin is a highly heritable trait, and important genetic gains can be obtained from the selection of high oleoresin yielder by traditional breeding methods [13]. We detected 26 terpenoids from the oleoresin in masson pines using GC/MS in this study. The major compounds of turpentine oil were alpha-pinene, beta-pinene, limonene and longifolene, and the major compounds of rosin were palustric acid, neoabietic acid, abietic acid, pimaric acid, dehydroabietic acid, isopimaric acid and sandaracopimaric acid in masson pines, which were consistent with the results from previous studies [27,28]. Among these terpenoids, the contents of tricyclene, camphene, β-myrcene, limonene, longipinene, longifolene, isopimaric and abietic acid revealed significantly difference between high and low oleoresin-yielding trees. In *P*. *halepensis*, myrcene, isopimaric and abietic acids were also reported to be differentiated in high oleoresin-yielding trees [20].

However, our understanding regarding key regulatory genes for the generation of oleoresin in conifers is still fragmental. In order to increase our knowledge of the genes related to the yield of oleoresin, we first analyzed the transcriptome of the secondary xylem that is the major secretion tissue of masson pines. We analyzed the transcriptome of masson pine, and obtained a rich resource of sequences. In the set of highly differentially expressed genes between high and low oleoresin-yielding trees, a number of genes were reported to participate in terpenoid backbone biosynthesis, terpenoid transport and defense response. GGPS, the isoprenyl diphosphate synthases belonging to a class of prenyltransferases, can catalyze the formation of geranylgeranyl diphosphate (GGPP) from isopentenyl diphosphate (IPP) and dimethylallyl diphosphate (DMAPP) via three successive condensation reactions [29,30]. GGPS genes were strongly up-regulated by both insect species and mechanical wounding in *Picea sitchensis* [31]. The up-regulation of GGPS genes was closely correlated with traumatic resin duct formation, thus producing higher amounts of monoterpenes, sesquiterpenes and diterpene resin acids [32]. In this study, the expression level of GGPS (comp99175_c0) was significantly different between high and low oleoresin-yielding masson pine trees, and had a higher expression in high oleoresin-yielding trees, suggesting the more efficient catalysis during the process of GGPP formation in high oleoresin-yielding trees.

The TPS enzyme generates one or more products using the substrate of GPP, FPP or GGPP by an electrophilic reaction [33–38]. Monoterpenes, sesquiterpenes and diterpenes are the products of GPP, FPP or GGPP, respectively. A large number of known TPS genes are induced for conifers in response to abiotic and biotic stimuli. In our study, the candidate gene of (-)-alpha/beta-pinene synthase (comp111620_c0) had higher expression in high oleoresin-yielding trees, while the candidate gene of tricyclene synthase (comp108110_c0) had higher expression in low oleoresin-yielding trees. Tricyclene, the product of tricyclene synthase, also had significantly different contents between high and low oleoresin-yielding genotypes. Alpha-pinene is a mixture of the isomers (+)-alpha-pinene and (-)-alpha-pinene. The content of (-)-alpha-pinene is approximately 10- to 20-fold higher than (+)-alpha-pinene in masson pines [39]. Although we found the significantly different expression of (-)-alpha/beta-pinene synthases between high and low oleoresin-yielding trees, there was no obvious difference in alpha-pinene using Mann-whitney tests.

GGPP formed by GPPS is the precursor of diterpenes, gibberellins, carotenoids and chlorophylls. Although the candidate gene GPPS had a higher expression level in high oleoresin-yielding trees, the content of diterpenes in oleoresin from high oleoresin-yielding trees was not higher in high oleoresin-yielding trees according to the statistical analysis using Mann-whitney tests. In contrary, oleoresin from high oleoresin-yielding trees had a higher content of monoterpenes and a lower content of diterpenes. After the stem is wounded, diterpene is transported by liquid monoterpenes and sesquiterpenes to the point of the wound, which can polymerize and seal the wound [7,8]. Therefore, we speculate that the increased diterpenes in low oleoresin-yielding trees can not only more rapidly seal the wound, but also can form physical barriers to prevent oleoresin outflowing, which will be the explanation of low oleoresin-yielding trees containing a higher content of diterpenes. The functional studies on the relevant genes are expected to be useful in the improvement of oleoresin yield and the resistance of the population of masson pines.

TPS enzymes producing oleoresin terpenoids have been localized to epithelial cells around resin ducts [35]. To the present, the transport mechanisms are unknown for the terpenoids from living epithelial cells to resin ducts in conifers [22]. The ABC transporters are confirmed to be participated in the active transport of dissimilar compounds using ATP hydrolysis energy [40]. For example, Jasinski et al. [41] have reported that *NpABC1* mediates the export of terpenoid from *Nicotiana plumbaginfolia* cells. In loblolly pine, Westbrook et al. [14] have also found that the expression of ABC transporter is regulated by oleoresin terpenoid. In our study, the variation in oleoresin yield is significantly associated with ABC transporters, which is consistent with the studies from Westbrook et al. [14]. Therefore, ABC transporters are also involved in the transport of conifer oleoresin.

In all differential expression genes associated to oleoresin yield, the candidate genes coding ERFs were involved. ERF containing a highly conserved DNA-binding domain plays an important role in regulating the expression of genes under external stresses [42]. In regulatory pathways of stress signal, ERFs can bind the GCC-box element presenting in many PR genes to control their expression [43]. In our study, the higher expression of ERFs was observed in high oleoresin-yielding trees after stem wounding.

To date, PR genes are commonly considered as the genes involved in systemically acquired resistance (SAR), and are coordinated with a wide range of plant defense genes responding to biotic and abiotic. PR5 family (thaumatin-like proteins) with low molecular weight can cause osmotic rupture [44]. In hybrid poplar, enhanced expression of PR5 is observed in phloem proteomic response to leaf wounding [45]. PR5 is also a hallmark of the resistant cultivar in interactions between *Hero A*-resistant tomatoes and cyst nematode [46]. In our study, PR5 is induced to a higher level in high oleoresin-yielding genotypes when compared with low oleoresin-yielding genotypes. Therefore, high oleoresin-yielding masson pine may have another characteristics of strong resistance, which has been proven in *P*. *taeda* by Storm et al. [9], wherein oleoresin flow is significantly higher in resistant trees than general population trees responding to the southern pine beetle. The deduction for masson pine will be verified in further studies.

## Conclusion

In this study, a comprehensive transcriptomic analysis from secondary xylems of masson pine was conducted, which provided a valuable resource for genetic and genomic study in the future. The candidate genes associated with oleoresin biosynthesis and the expression profiles of high and low oleoresin-yielding genotypes were characterized by *de novo* assembly and DEGs analysis. GO enrichment analysis of DEGs revealed that multiple biological pathways including light signaling response, photosynthesis and lipid metabolism were related to the high yield of oleoresin. Further analysis of candidate genes involved in terpenoid backbone biosynthesis revealed that GGPS and TPS enzymes of (-)-alpha/beta-pinene, and tricyclene synthase were related to the high yield of oleoresin. These results coordinated well with GC/MS data that the products of tricyclene synthases were more enriched in high-yielding genotype than low-yielding genotype. Moreover, DEGs of PRs, ABC transporters, non-specific lipid-transfer protein-like protein, phosphomethylpyrimidine synthase and ERFs might be important for the high yield of oleoresin. Overall, our work provided new insights into the molecular mechanisms for oleoresin production, which may lay a solid foundation for expediting the breeding of high-yield oleoresin pines.

## Materials and Methods

### Plant materials

The experiments were conducted using twenty-seven-year-old masson pine clones cultivated in the first generation seed orchard established in 1986 by Laoshan Forest Farm, Chunan County, Zhejiang, China. The clones were propagated by grafting and no any relationships were existed between their ortets. In 2012, the yield of oleoresin was measured using the bark streak method of wounding for oleoresin tapping [15]. No stimulant to induce oleoresin flow was applied. The yield of oleoresin was calculated as the yield of the individual per day per cm streak length in grams to adjusting stem diameter influence on oleoresin yield. Based on the measurement, 35 high oleoresin-yielding clones (more than 5.0 g d^-1^ cm^-1^) and 35 low oleoresin-yielding clones (less than 1.5 g d^-1^ cm^-1^) were tapped the xylem oleoresin using the traditional bark streak method of wounding to analyze the compositions of terpenoids. The fresh oleoresin was collected using 1.5 mL polyethylene tube for each sample and the tube was hermitically closed and refrigerated until the analysis.

From these clones, 10 high oleoresin-yielding clones and 10 low oleoresin-yielding clones were selected for the analysis of transcriptome and DEGs. The low oleoresin-yielding clones were served as the control. Three ramets for each clone were selected as three biological replicates. No protected species were involved in this study. After the removal of the bark, phloem, cambium and surface layer of tapping streak last year, approximately 5 mm deep fresh secondary xylem tissues adjoining cambium were harvested from sample trees. These samples were put into liquid nitrogen immediately in field and then stored at -80°C for RNA extraction.

### Analysis of oleoresin compositions

For quantitative analysis, the samples for terpenoid identification using gas chromatography-mass spectrometry (GC/MS) were prepared according to Karanikas’ method [20].

Subsequently, terpenoids of masson pine were detected by GC/MS with an Agilent 6890N gas chromatography coupled with a HP-5MS column (ID: 0.25 mm, length: 30 m, film thickness: 0.25 μm), and an Agilent 5975B mass spectrometer. For GC, the program of oven temperature was 60°C for 2 min, increasing 2°C/min to 80°C, 80°C for 5 min, subsequent increase rate of 4°C/min until 280°C and 280°C for 5 min. Helium was used as the carrier gas at a flow rate of 1 mL/min, injector temperature was set up at 260°C, injection volume was 1 μL with a split ratio of 1:50. Electron ionization mass spectrometry analysis was carried out with 70 eV electron energy, 230°C ion source and 280°C connection part temperature. Terpenoids were identified by matching fragmentation of mass spectra with NIST08. In addition, experimental retention indices were also used to match with reference compounds. The concentration of each terpenoid was expressed as the mass of a compound in oleoresin per gram. SPSS 18.0 statistical package was used to perform the statistical analysis. The significant difference between high and low oleoresin-yielding clones was determined by non-parametric Mann-Whitney test.

### RNA isolation

Total RNA from each sample of twenty high and low oleoresin-yielding clones was separately extracted using Plant RNA kit of RN38 EASYspin plus (Aidlab Biotech, Beijing, China) containing DNA digestive enzyme. The concentration of total RNA was detected using Ultrasec 2100 pro UV/Visible Spectrophotometer, and the integrity of total RNA was detected using 1% agarose gel electrophoresis. Then, the integrity of RNA was also tested using an Agilent 2100 Bioanalyzer before proceeding. The RNA with concentration more than 400 ng/μL, OD260/280 ranging from 1.8 to 2.2, OD260/230 over 2.0, 28S/18S no less than 1.5 and RIN over 8.0, was defined as high quality RNA. Subsequently, equal quantity of RNA from one ramet of each high oleoresin-yielding clone was pooled for cDNA synthesis. For low oleoresin-yielding clones, the RNAs were pooled using the same method. A total of six RNA samples were obtained, including three biological replicates for high and low oleoresin-yielding genotypes, respectively.

### Transcriptome sequencing and assembly of masson pine

To obtain a comprehensive list of transcripts, equal amounts of high-quality RNA from six RNA samples were pooled together. Then, total RNA was delivered to Biomarker Technology Company (Beijing, China) to construct cDNA library and sequencing. The cDNA library was paired-end sequenced on the Illumina HiSeq 2000 sequencing platform, and 2*×* 101 bp reads were yielded from either end of a cDNA fragment.

After filtering the raw reads, we assembled the high quality reads into contigs using Trinity method [47]. Then, the transcripts were assembled with the paired-end information of contigs. Finally, the longest transcripts from the potential alternative splicing transcripts were selected as the unigenes. The data has been submitted to NCBI, and the accession number is SRP050025.

### Functional annotations and classifications

Functional annotations were carried out by matching sequences with public databases. All unigenes were matched with NCBI Nr database, Swiss-Prot database and COG database using BLASTx with the E-value of less than 10^-5^. GO classification was performed using WEGO software (http://wego.genomics.org.cn/cgibin/wego/index.pl). Pathway assignments were achieved from KEGG web server. RPKM was used to represent the expression abundance of the unigenes.

### Differentially expressed genes and GO enrichment

All high quality reads were aligned to the assembled transcripts above using Bowtie, allowing no more than one nucleotide mismatch. In order to compare the expression abundance among six samples, tags were normalized into RPKM values. The differential expression of genes was analyzed by the edgeR package (Version 3.2.4) in BioConductor (release 2.12, R version 2.15.0) [48]. The right-sided hypergeometric enrichment test was performed at a medium network specificity selection and p-value correction was performed using the Benjamini-Hochberg method. The GO terms with p-value less than or equal to 0.05 were considered as the significant enrichment. The biological process of enriched GO terms was visualized by using ReviGO [26]. And the relatives of enriched GO terms were identified and plotted by using Matlab Bioinformatics toolbox (Mathworks Inc.).

### Quantitative RT-PCR analysis

The RNA samples used in qRT-PCR and DGE experiments were identical. The primer pairs (S8 Table) for representative candidate genes were designed using Primer 3.0 software with the optimal Tm at 58–62°C, primer length of 19–21 nucleotides, PCR product size of 120–200 bp and GC content of 45–55%.

Quantitative RT-PCR was run in a 7300 Real Time PCR System (Applied Biosystems, CA, USA) with the SYBR green detection method to verify the DGE results. The cDNA was amplified in a total volume of 20 μL including 0.4 μL ROX reference dye, 2 μL cDNA, 0.4 μL primers and 10 μL SYBR Premix Ex Taq. The program of PCR reactions was 95°C for 10 s, next 40 cycles of 95°C for 5 s, 58°C for 30 s and 72°C for 30 s. Three technical replicates for each of three biological replicates were performed. The transcript profiles were normalized using the reference gene of elongation factor 1-alpha, and the relative expression levels of candidate genes were calculated with the 2^-ΔΔCt^ method [49].

## Supporting Information

S1 Fig The profile for identified oleoresin terpenoids in masson pines using GC/MS analysis. The oleoresin from 1102-3-2 clone was as a representative to show the profile for identifying oleoresin terpenoids in masson pine using GC/MS analysis.
(TIF)

S2 Fig Functional annotation of assembled sequences based on gene ontology (GO) categorization. The unigenes were annotated in three categories: cellular components, molecular functions and biological processes.
(TIF)

S3 Fig Clusters of orthologous group (COG) classification. Totally 9,990 of 84,842 sequences with Nr hits were grouped into 25 COG classifications.
(TIF)

S1 Table Summary of Illumina transcriptome assembly in masson pine. (DOC)

S2 Table Functional annotation of the masson pine transcriptome. (DOC)

S3 Table Details for 35,353 unigenes annotated in the transcriptome of xylem in masson pine. (XLS)

S4 Table Read matched sequences in the transcriptome. (DOC)

S5 Table Expression of 649 DEGs in three biological replicates for high-yielding genotypes (H1, H2 and H3) and low-yielding genotypes (L1, L2 and L3). (XLS)

S6 Table The significantly enriched GO terms. (XLS)

S7 Table The expression of key candidate genes in high and low oleoresin-yielding trees (three biological replicates). (DOC)

S8 Table Primers for quantitative real-time PCR. Primers were designed from the sequences of masson pine transcriptome library by using Primer Premier 3.0.
(DOC)
