# Supplementary figures and images for: Genome-Wide Identification of Differentially Expressed Genes Associated with the High Yielding of Oleoresin in Secondary Xylem of Masson Pine (Pinus massoniana Lamb) by Transcriptomic Analysis
Source: PLoS One. 2015 Jul 13;10(7):e0132624. doi: 10.1371/journal.pone.0132624 (PMC4500461; doi:10.1371/journal.pone.0132624)

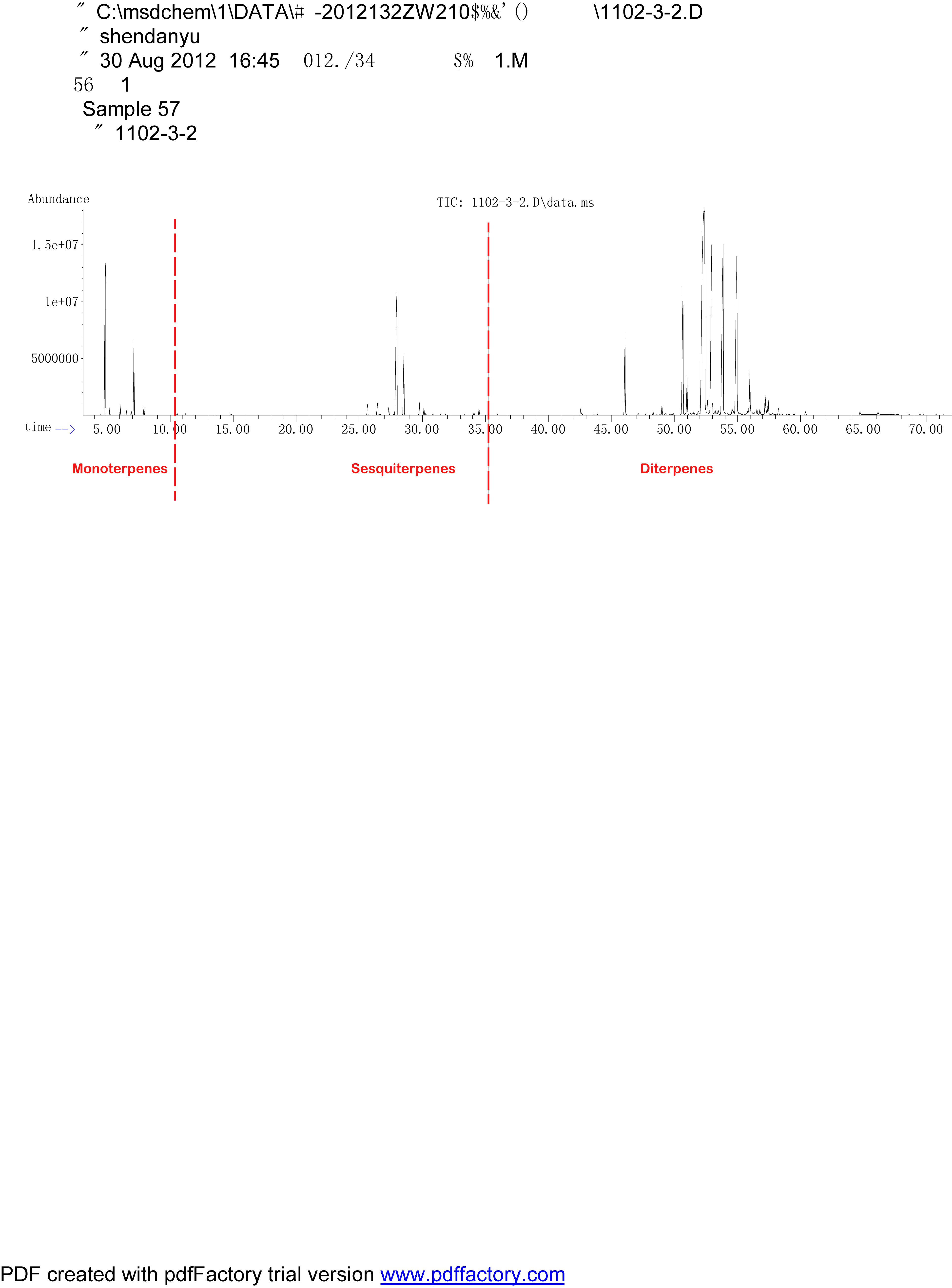

Supplement: S1 Fig — The oleoresin from 1102-3-2 clone was as a representative to show the profile for identifying oleoresin terpenoids in masson pine using GC/MS analysis. (TIF) [file pone.0132624.s001.tif]

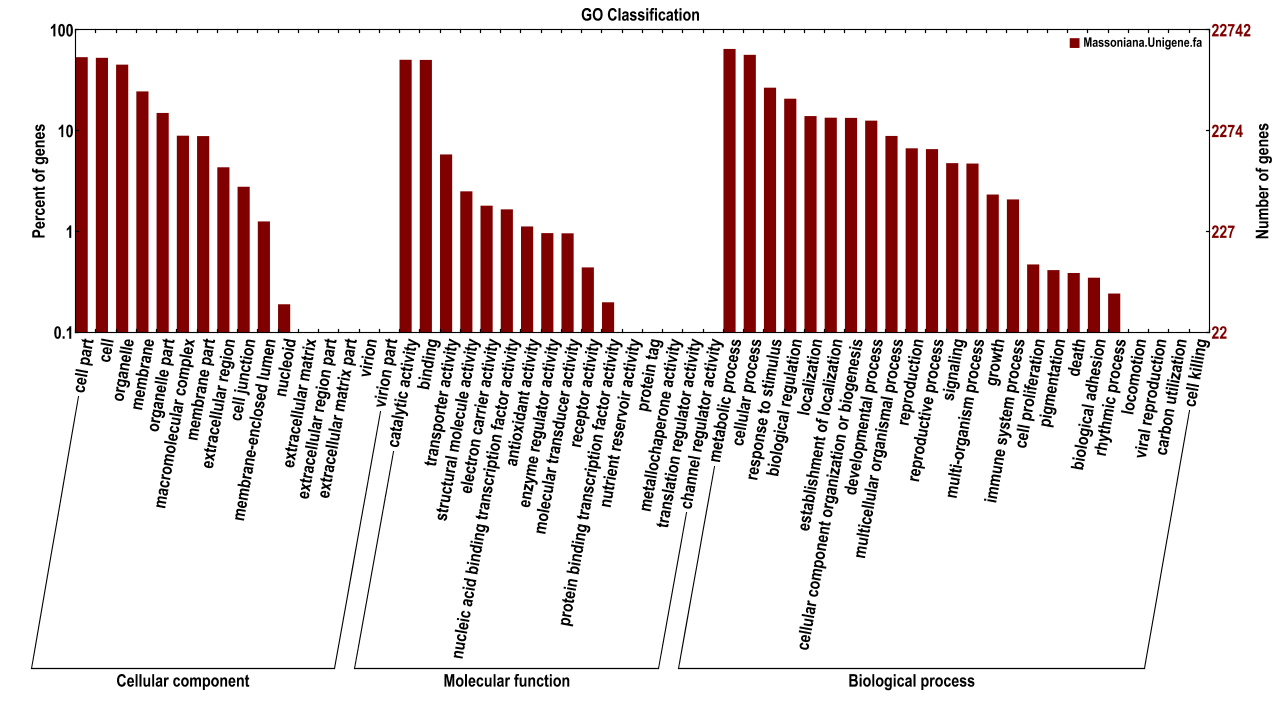

Supplement: S2 Fig — The unigenes were annotated in three categories: cellular components, molecular functions and biological processes. (TIF) [file pone.0132624.s002.tif]

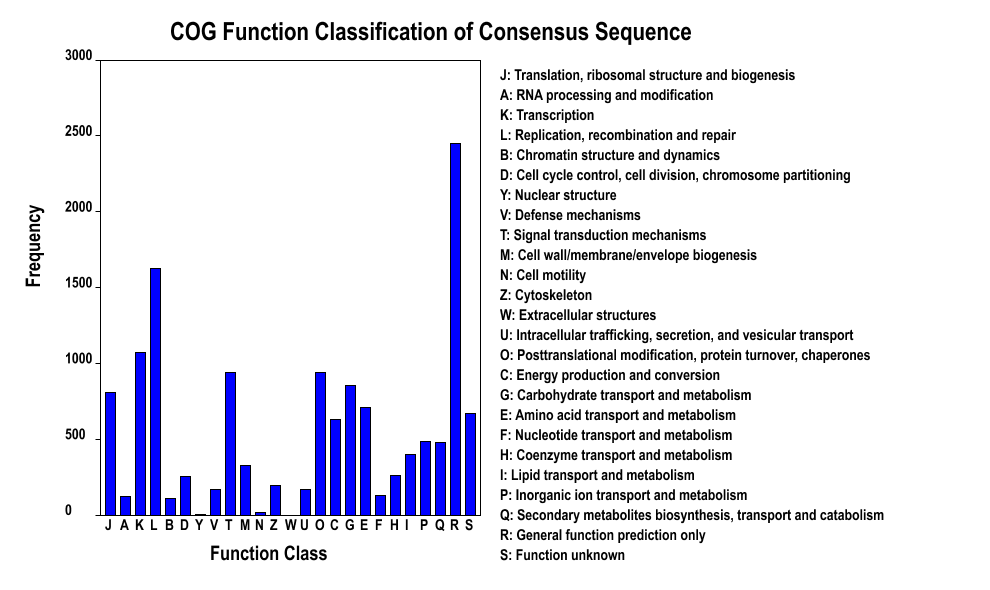

Supplement: S3 Fig — Totally 9,990 of 84,842 sequences with Nr hits were grouped into 25 COG classifications. (TIF) [file pone.0132624.s003.tif]
