# Supplementary material for: Genome-Wide Identification of Differentially Expressed Genes Associated with the High Yielding of Oleoresin in Secondary Xylem of Masson Pine (Pinus massoniana Lamb) by Transcriptomic Analysis
Source: PLoS One. 2015 Jul 13;10(7):e0132624. doi: 10.1371/journal.pone.0132624 (PMC4500461; doi:10.1371/journal.pone.0132624)
Supplement: S1 Table — (DOC) [file pone.0132624.s004.doc]

**Table S1**. Summary of Illumina transcriptome assembly for masson pine.

| **Nucleotide length (bp)** | **Contigs** | **Transcripts** | **Unigenes** |
| --- | --- | --- | --- |
| 0-300 | 3579277 | 41506 | 34011 |
| 300-500 | 29147 | 31236 | 22703 |
| 500-1000 | 16127 | 25308 | 12415 |
| 1000-2000 | 10157 | 22830 | 9186 |
| 2000+ | 6592 | 16619 | 6527 |
| Total number | 3641300 | 137499 | 84842 |
| Total length | 259141153 | 124348385 | 58578462 |
| N50 length | 72 | 1671 | 1291 |
| Mean length | 71.17 | 904.36 | 690.44 |
