# Supplementary material for: Genome-Wide Identification of Differentially Expressed Genes Associated with the High Yielding of Oleoresin in Secondary Xylem of Masson Pine (Pinus massoniana Lamb) by Transcriptomic Analysis
Source: PLoS One. 2015 Jul 13;10(7):e0132624. doi: 10.1371/journal.pone.0132624 (PMC4500461; doi:10.1371/journal.pone.0132624)
Supplement: S2 Table — (DOC) [file pone.0132624.s005.doc]

**Table S2.** **Functional annotation of the masson pine transcriptome.**

| **Annotated databases** | **All sequences** | **≥ 300 bp** | **≥ 1000 bp** |
| --- | --- | --- | --- |
| COG_Annotation | 9,990 | 2,706 | 5,761 |
| GO_Annotation | 27,147 | 9,561 | 11,728 |
| KEGG_Annotation | 6,465 | 1,903 | 3,454 |
| Swissprot_Annotation | 22,748 | 8,215 | 9,846 |
| nr_Annotation | 35,200 | 13,036 | 14,065 |
| Total | 35,353 | 13,103 | 14,075 |
