# Supplementary material for: Genome-Wide Identification of Differentially Expressed Genes Associated with the High Yielding of Oleoresin in Secondary Xylem of Masson Pine (Pinus massoniana Lamb) by Transcriptomic Analysis
Source: PLoS One. 2015 Jul 13;10(7):e0132624. doi: 10.1371/journal.pone.0132624 (PMC4500461; doi:10.1371/journal.pone.0132624)
Supplement: S4 Table — (DOC) [file pone.0132624.s007.doc]

**Table S4. Reads matched sequences in the transcriptome**.

| **Samples** | **Mapped Reads** | **Uniq mapped Reads** | **Multi mapped Reads** |
| --- | --- | --- | --- |
| H-1 | 8,809,960 (81.96%) | 8,176,257 (92.81%) | 633,703 (7.19%) |
| H-2 | 7,863,893 (81.84%) | 7,318,833 (93.07%) | 545,060 (6.93%) |
| H-3 | 8,238,323 (81.54%) | 7,658,502 (92.96%) | 579,821 (7.04%) |
| L-1 | 8,316,147 (82.16%) | 7,736,566 (93.03) | 579,581 (6.97%) |
| L-2 | 8,518,326 (82.05%) | 7,905,342 (92.80%) | 612,984 (7.20%) |
| L-3 | 10,080,682 (81.48%) | 9,374,336 (92.99%) | 706,346 (7.01%) |
