# Supplementary material for: Genome-Wide Identification of Differentially Expressed Genes Associated with the High Yielding of Oleoresin in Secondary Xylem of Masson Pine (Pinus massoniana Lamb) by Transcriptomic Analysis
Source: PLoS One. 2015 Jul 13;10(7):e0132624. doi: 10.1371/journal.pone.0132624 (PMC4500461; doi:10.1371/journal.pone.0132624)
Supplement: S7 Table — (DOC) [file pone.0132624.s010.doc]

**Table S7.** The expression of key genes in high- and low-yielding oleoresin trees (three biological replicates)

| **Annotation results** | **Seq_ID** | **High-yielding oleoresin trees** | | | **Low-yielding oleoresin trees** | | | **Regulated** |
| --- | --- | --- | --- | --- | --- | --- | --- | --- |
|  |  | **H1** | **H2** | **H3** | **L1** | **L2** | **L3** |  |
| GGPs | comp99175_c0 | 4.64 | 5.42 | 4.60 | 0.57 | 0.74 | 0.47 | up |
| Tricyclene synthase | comp108110_c0 | 0.08 | 0.00 | 0.00 | 2.29 | 2.48 | 2.18 | down |
| (-)-alpha/beta-pinene synthase | comp111620_c0 | 9.68 | 9.89 | 9.43 | 0.62 | 0.91 | 0.51 | up |
| Phosphomethylpyrimidine synthase | comp125324_c1 | 20.65 | 19.33 | 8.99 | 36.03 | 45.39 | 37.48 | down |
| ABC transporter | comp126204_c0 | 9.21 | 11.31 | 11.11 | 20.00 | 23.31 | 20.76 | down |
|  | comp96152_c0 | 0.74 | 0.84 | 0.80 | 0.00 | 0.00 | 0.00 | up |
| Non-specificlipid-transfer protein-like protein | comp123400_c0 | 16.94 | 18.26 | 20.77 | 10.56 | 10.04 | 12.87 | up |
| ERFs | comp102852_c0 | 2.82 | 3.17 | 2.56 | 0.69 | 0.00 | 0.00 | up |
|  | comp121364_c0 | 13.80 | 10.02 | 11.72 | 0.16 | 0.08 | 0.27 | up |
| PR5 (Thaumatin-like) | comp111031_c0 | 18.28 | 18.77 | 16.94 | 0.96 | 0.47 | 0.40 | up |
| PR9 (Peroxidase) | comp121150_c0 | 20.42 | 17.68 | 16.24 | 2.61 | 2.53 | 2.26 | up |
| TMV resistance protein | comp123469_c1 | 2.39 | 2.17 | 2.46 | 1.61 | 1.40 | 1.21 | up |
|  | comp122350_c2 | 4.96 | 5.58 | 3.78 | 1.25 | 0.54 | 1.14 | up |
