# Supplementary material for: Genome-Wide Identification of Differentially Expressed Genes Associated with the High Yielding of Oleoresin in Secondary Xylem of Masson Pine (Pinus massoniana Lamb) by Transcriptomic Analysis
Source: PLoS One. 2015 Jul 13;10(7):e0132624. doi: 10.1371/journal.pone.0132624 (PMC4500461; doi:10.1371/journal.pone.0132624)
Supplement: S8 Table — Primers were designed from the sequences of masson pine transcriptome library by using Primer Premier 3.0. (DOC) [file pone.0132624.s011.doc]

**Table S8. Primers for relative quantitative realtime PCR. Primers were designed from the sequences of masson pine transcriptome library by using Primer Premier 3.0.**

| **ID** | **Gene** | **Primers** |
| --- | --- | --- |
| comp99175_c0 | GGPS | F: GATGCCCTAATCTCGTGGAA R: TGCATTCTATTGCTCCGTTG |
| comp108110_c0 | Tricyclene synthase | F: AGCCAGGTGGTACATCCAAG R: AGCATTCCATCAGCCTGTCT |
| comp111620_c0 | (-)-alpha/beta-pinene synthase | F: AAATCGTGTGTGTCCCTGCA R: TTCACGGTAAGCGAGTTCCC |
| comp96152_c0 | ABC transporter | F: CCGAAGTAATCCTCCACGAA R: CCGAAGTAATCCTCCACGAA |
| comp125324_c1 | Phosphomethylpyrimidine synthase | F: TAAAGGGCATCCATTTGCTC R: TGGAACAAAAGTGGGCTACC |
| comp123400_c0 | Non-specificlipid-transfer protein-like protein | F: TTCACGTCAACTGCAGGAAC R: ACGGCGCTAAAGTAACGAGA |
| comp121364_c0 | ERFs | F: AGTGGGACGGGCGAAATTAA R: ACCTGGGAGATTTTCAGCGG |
| comp111031_c0 | PR-5 family (thaumatin-like proteins) | F: TGAAGCTGCAACATTTGTGA R: AAATCTGCCTGCACTTGTCC |
| comp121150_c0 | PR-9 family (peroxidase) | F: ACCAATTTGCTCTCCACAGG R: TCACTATTTCCTCGGCCTTG |
